# Feeding Difficulties in Late Preterm Infants and Their Impact on Maternal Mental Health and the Mother–Infant Relationship: A Literature Review

**DOI:** 10.3390/nu15092180

**Published:** 2023-05-03

**Authors:** Giulia Vizzari, Daniela Morniroli, Arianna D’Auria, Paola Travella, Elena Bezze, Patrizio Sannino, Serena Rampini, Paola Marchisio, Laura Plevani, Fabio Mosca, Maria Lorella Giannì

**Affiliations:** 1 Fondazione IRCCS Ca’ Granda Ospedale Maggiore Policlinico, NICU, 20122 Milan, Italy; giulia.vizzari@gmail.com (G.V.); laura.plevani@policlinico.mi.it (L.P.); fabio.mosca@unimi.it (F.M.); 2 Department of Clinical Sciences and Community Health, University of Milan, 20122 Milan, Italy; daniela.morniroli@unimi.it (D.M.); p.travella@outlook.it (P.T.); 3 Fondazione IRCCS Ca’ Granda Ospedale Maggiore Policlinico, Direzione Professioni Sanitarie, 20122 Milan, Italy; elena.bezze@policlinico.mi.it (E.B.); patrizio.sannino@policlinico.mi.it (P.S.); serena.rampini@policlinico.mi.it (S.R.); 4 Fondazione IRCCS Ca’ Granda Ospedale Maggiore Policlinico, Pediatric Highly Intensive Care Unit, 20122 Milan, Italy; paola.marchisio@unimi.it; 5 Department of Pathophysiology and Transplantation, University of Milan, 20122 Milan, Italy

**Keywords:** late preterm infants, feeding difficulties, breastfeeding, maternal health, mother–infant relationship

## Abstract

Late preterm infants constitute the largest subset of premature infants and are more likely to experience feeding issues leading to delayed oral feeding independence and low breastfeeding rates. Considering the increased parental concern about their infants’ nutrition and growth, we performed a literature review to provide an update on the feeding challenges faced by late preterm infants and the impact of these issues on maternal mental health and the mother–infant relationship. Based on our findings, late preterm infants have a high prevalence of feeding difficulties which need to be addressed by targeted support interventions to promote breastfeeding success and the establishment of a harmonious dyadic interaction between the mother and her infant, all of which contribute to the prevention of altered feeding behavior later in life. There is still a need for additional research to develop a standardized and shared strategy that can be proven to be effective. Should this be accomplished, it will be possible to offer appropriate support for mothers, encourage the oral skills and maturation of late preterm infants, and improve the relationship quality within the dyad.

## 1. Introduction

Late preterm infants are born between 34 and 36 weeks of gestation. They constitute the largest subset of premature infants and may experience some of the same difficulties and issues as extremely premature infants. [1]. An increasing amount of evidence indicates that late preterm infants are more likely to experience feeding issues that may persist through childhood [2,3]. Owing to their lower oromotor tone, poor sucking-swallowing-breathing coordination, and disturbed sleep-wake cycles, they have prolonged nasogastric tube feeding and delayed oral feeding independence [4,5,6]. In addition, late preterm newborns have an immature gastrointestinal function, with a higher incidence of gastroesophageal reflux than their full-term counterparts [6]. All these variables impede the successful initiation and continuation of breastfeeding, resulting in its early cessation [6,7]. Hence, the breastfeeding rates of late preterm infants are lower than those of full-term infants despite the widely acknowledged health benefits for infants and mothers [8,9]. Moreover, feeding difficulties are among the most common reasons for hospital readmission of these infants after discharge [10].

Due to the difficulty of independent oral feeding for late preterm infants, parents are concerned about their infants’ nutrition and growth [11,12]. Hence, the increased parental concern may also negatively affect the parent’s mental health and the mother–infant bond [11].

Our review seeks to offer an update on the feeding challenges faced by late preterm infants and the impact of these issues on maternal mental health and the mother–infant relationship.

## 2. Materials and Methods

We performed a bibliographic search in the PubMed, Cinahl, and PsycINFO databases in July 2022. The following search strings were entered: ((“late preterm” OR “near term”) AND (feed* OR nutrition OR diet)) AND (mother*) in PubMed using key words; (((“Infant, Premature”[Mesh]) OR (“Premature Birth”[Mesh])) AND ((“Diet, Food, and Nutrition”[Mesh] OR “Nutrition Disorders”[Mesh]) OR “Feeding and Eating Disorders”[Mesh])) AND ((“Mother-Child Relations/psychology”[Mesh]) OR (“Mothers/psychology”[Mesh])) in PubMed, searching by term Thesaurus (Mesh); ((MH “Childbirth, Premature”) OR (MH “Infant, Premature”)) AND ((MH “Mother-Child Relations”) OR (MH “Mothers+”)) AND ((MH “Infant Nutrition+”) OR (MH “Infant Nutrition Disorders”)) AND (psychology or psychological or emotions or emotionality or emotional or feeling or psychosocial or relation or relationship) in Cinahl; exp Premature Birth/and exp Mother Child Relations/AND (exp Breast Feeding/or exp Diets/OR exp Bottle Feeding/or exp Feeding Disorders OR exp Nutrition/ OR exp Eating Behavior/or exp Food/or exp Food Intake/) in PsycINFO selecting Multi-field search. The grey literature was not reviewed.

**Figure 1:**
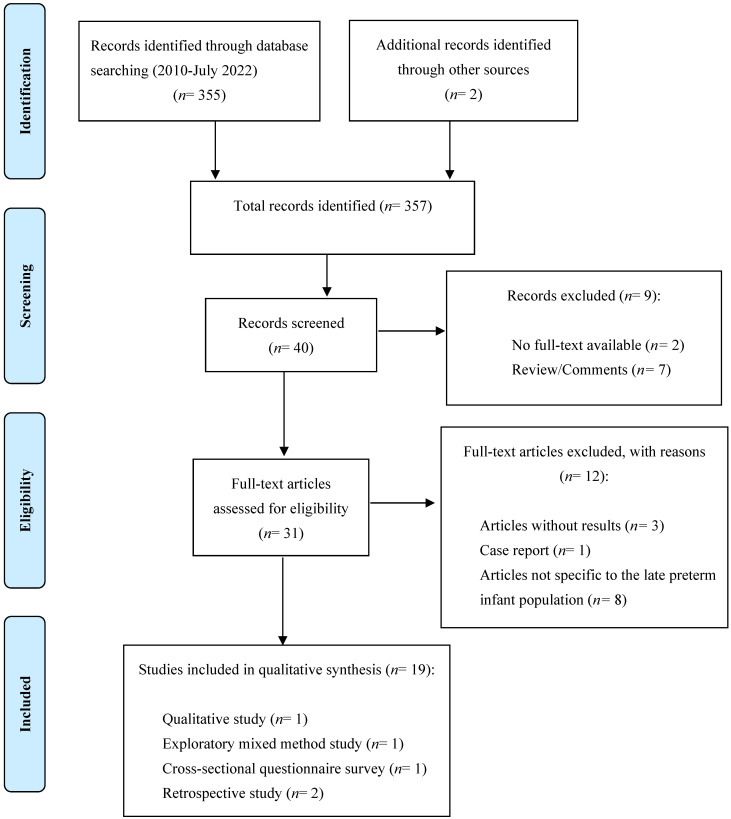
PRISMA 2009 research method flowchart.

The bibliography search outcome led us to 355 publications. Following the revision of the references of the retrieved articles, two additional studies were identified (Figure 1), leading to a final count of 357 publications. Among them, 317 were excluded based on the title or the abstract because they were not pertaining. A total of 40 articles were screened, and nine were further excluded. Two researchers independently searched, screened, and identified studies for eligibility. A third additional researcher addressed any discrepancy to reach a consensus.

To be included, studies had to be either observational, clinical trials, qualitative or mixed method studies, published between the 1st of January 2010 and the 21st of July 2022, with a full text available in English, and to investigate the incidence of feeding difficulties in late preterm infants and the effect of feeding difficulties on maternal mental health and the mother–infant relationship. Publications regarding newborns with genetic syndromes and/or defects of the face, jaw, mouth, and gastrointestinal tract, neurodevelopmental impairment due to severe neonatal comorbidities, such as hypoxic-ischemic encephalopathy, neonatal asphyxia, intracranial hemorrhage, or who had undergone abdominal surgery were excluded. Publications that were not specific to the late preterm infant population were also removed, as were meta-analyses, reviews, editorial comments, conference abstracts, and unpublished dissertations. Finally, 19 studies were selected: 17 observational studies, one qualitative study, and one mixed exploratory study.

The literature search process is shown in Figure 1.

## 3. Results

### 3.1. Feeding Difficulties in Late Preterm Infants

A total of 15 studies, eight conducted in North America, six in Europe, and one in Asia, addressed the occurrence of feeding difficulties in late preterm infants. Among them, nine were prospective observational studies, two retrospectives, two cross-sectional, one qualitative, and one mixed method. The results from the included studies indicate that breastfeeding rates in late preterm infants are lower than those of term infants due to the occurrence of feeding difficulties mostly related to their immature physiologic functions and/or oromotor dysfunctions, particularly at the lowest gestational ages. An improvement in breastfeeding rates through the first weeks after hospital discharge has been reported, reflecting the time late preterm infants need to achieve competency in feeding.

Gianni et al. [13] performed a cross-sectional questionnaire survey including 92 mothers of late preterm infants admitted to level I and II of care. The authors reported that at discharge, 94% of infants were fed any human milk. However, only 43% of them were exclusively human milk fed. Dosani et al. [14] conducted an exploratory mixed method study, enrolling a convenient sample of 122 mothers of late preterm infants. The authors found that the exclusive breastfeeding rate at 6–8 weeks postpartum was 14%. Moreover, they interviewed 11 mothers and ten nurses who reported that breastfeeding late preterm infants is challenging mainly due to the lack of effective coordination of sucking, swallowing, and breathing. Kair et al. [15] conducted a qualitative study to assess the breastfeeding experience of late preterm mothers. Breastfeeding represented a positive bonding experience. However, mothers reported milk supply concerns, negative experiences with breast pumping, and feelings of failure. In line with the previous findings, Crippa et al. [16] conducted an observational study on 189 late preterm infants admitted to level I of care. The authors reported an exclusive breastfeeding rate at discharge of 16.8%, which increased to 40.3% at 15 days, reflecting that late preterm infants needed more time to become competent in breastfeeding skills. Nagulesapillai et al. [17], in their community-based prospective pregnancy cohort, including 137 late preterm mothers interviewed at four months after delivery, demonstrated that being born late preterm was an independent risk factor for the occurrence of breastfeeding difficulties related to the baby (OR 1.72, 95% CI 1.24–2.38). Accordingly, DeMauro et al. [18] interviewed parents of 571 late preterm infants. They reported that at three months, 27% showed symptoms such as choking and spitting compatible with oromotor dysfunction (17%) and avoidant feeding behavior (29%), decreasing to 4% and 12% at one year, respectively.

Lee et al. [19], in their observational study including 106 late preterm infants hospitalized in the neonatal intensive care unit, reported a similar trend of breastfeeding rates, which increased from 5.7% at the end of the first week to 19.8% at the 12th week. Moreover, the authors found that 43.4% of the mothers reported feeding difficulties. McDonald et al. [20] performed a prospective community-based cohort study including 77 mothers of late preterm infants. The authors reported that late preterm mothers experienced more frequent breastfeeding difficulties and earlier discontinuation of breastfeeding at four months than full-term mothers (69.3 vs. 81.7%, respectively).

Jonsdottir et al. [21] conducted a cohort study, including 122 late preterm mothers and 269 term mothers. They found that late preterm infants were less likely to be exclusively breastfed than term ones during the first week after discharge and at one month. Following these findings, the authors compared breastfeeding duration through the first year of life between a cohort of 129 late preterm infants and 277 term infants [22]. They reported that late preterm infants were breastfed significantly shorter than term infants (median time seven months vs. nine months, respectively).

The risk of presenting feeding difficulties is greater the lower the gestational age at birth. Medoff Cooper et al. [23], in their multicenter prospective study, showed that, among the 802 enrolled late preterm infants, feeding difficulties occurred in 40.6% of cases, with a higher percentage in infants born at 34 weeks of gestational age compared with babies born at 35 and 36 weeks of gestational age (61% vs. 42 and 35%, respectively). Accordingly, Hellmeyer et al. [24] investigated the occurrence of feeding difficulties in 893 late preterm infants and reported a higher incidence of feeding difficulties in 35 weeks than in 36 ones (60.6% vs. 50%). Gianni et al. [4] conducted a retrospective study investigating the need for nutritional support among the 1768 late preterm infants born in 2011–2013 at the authors’ Institution. The authors found that 33.5% of the enrolled infants received nutritional support; among these, 2.6% required tube feeding, with the highest percentage among the infants born at 34 weeks of gestational age as compared to infants born at 35 and 36 weeks of gestational age (5.3% vs. 2.8% and 1.3%, respectively). Consistently, Lau et al. [25] investigated the oral infants’ feeding skills of 48 late preterm infants at the time of their first oral feed. They found that infants 34 weeks of gestational age had poorer performance than their 35-week counterparts. Demirci et al. [26] highlighted that, although the breastfeeding rates among the enrolled late preterm infants were lower than those of full-term ones, mothers who gave birth at 35 and 36 weeks were more likely to initiate breastfeeding than mothers of a 34-week infant.

Table 1 reports the main findings of the studies included in the review investigating feeding difficulties in late preterm infants.

### 3.2. Effect of Feeding Difficulties on Maternal Mental Health

A total of four prospective observational studies, three conducted in Europe and one in North America, addressed the effect of feeding difficulties on maternal mental health. Based on the results of the included studies, mothers of late preterm infants experience a higher level of anxiety, depression, and stress than mothers of full-term infants, which in turn negatively affects breastfeeding success.

In their prospective community-based cohort, McDonald et al. [20] included 77 mothers of late preterm infants and reported that late preterm mothers were at higher risk of having anxiety symptoms four months after delivery than full-term mothers (OR 2.07, CI 1.08; 3.98). Zanardo et al. [27] performed a prospective case-control study in 42 late preterm mothers matched with 42 mothers of full-term newborns to assess their psychological distress in the postpartum period. The late preterm mothers showed a significantly higher state of anxiety, depression, and stress than full-term mothers, as indicated by the scores gained according to the State-Trait Anxiety Inventory (STAI), the Edinburgh Postnatal Depression Scale (EPDS), and the Psychological Stress Measure (PSM) (trait anxiety 45.8 ± 10.1 vs. 39 ± 6.1, *p* < 0.02; state anxiety, 49.5 ± 9 vs. 42.6 ± 5.3, *p* < 0.002; EPDS, 9.5 ± 4.5 vs. 6.3 ± 3.9, *p* < 0.0008; PSM, 46.5 + 5.9 vs. 38.9, *p* < 0.001, respectively). Moreover, the psychological distress experienced by late preterm mothers was the most significant independent risk factor for early cessation of breastfeeding. In a following case-control study, the authors [28] compared the maternal personality profile and attitudes towards lactation between 30 late preterm mothers and 60 full-term mothers. They reported that late preterm mothers experienced deep stress according to the Luscher Color Test general interpretation.

Jonsdottir et al. [22] found that 53%, 44%, and 44% of the 129 late preterm mothers enrolled in their cohort study were worried about their infant’s nutrition at 1, 4, and 12 months, respectively. Moreover, the authors reported that at four months, 18% of the late preterm mothers scored ≥ 13 at Edinburgh Postnatal Depression Score (EPDS) vs. 8% of full-term mothers (*p* < 0.01).

Table 1 reports the main findings of the studies included in the review investigating the effect of feeding difficulties on maternal mental health and the maternal-infant relationship.

### 3.3. Effect of Feeding Difficulties on Maternal–Infant Relationship

A total of three prospective observational studies conducted in Europe addressed the effect of feeding difficulties on the maternal–infant relationship. The results of the included studies highlighted that mothers of late preterm mothers show an altered bonding which is associated with the reduction of breastfeeding rates in the months following hospital discharge. A direct correlation was also reported between maternal self-efficacy and the degree of adaptation to infants’ breastfeeding behavior and success.

Zanardo et al. [28] aimed to compare the mother-to-infant bonding attitude among 30 mothers of late preterm infants and 60 mothers of full-term infants by administering the Mother-to-Infant Bonding Scale. The authors found that mothers of late preterm infants had significantly lower bonding as indicated by the scoring of dislike and disappointment. Moreover, a significant negative association was found between the bonding alteration of late preterm mothers and reduced breastfeeding rates from discharge to the following six postnatal months. In line with these findings, Gerhardsson et al. [29] highlighted the virtuous circle between maternal self-efficacy and successful breastfeeding. The authors assessed maternal self-efficacy in a cohort of 105 late preterm mothers by administering the Breastfeeding Self-Efficacy Scale-Short Form and reported a direct association between the degree of self-efficacy and the degree of adaptation to their infant’s breastfeeding behavior. The authors further assessed the association of the degree of maternal self-efficacy on breastfeeding duration, highlighting that, among the enrolled late preterm mothers, those exclusively breastfeeding at 40 weeks and three months of corrected age had higher scores according to the Breastfeeding Self-Efficacy Scale than those who did not [30].

## 4. Discussion

The most prevalent feeding issues were challenging breastfeeding establishment and maintenance, delayed oral feeding, poor coordination in sucking-swallowing-breathing reflexes, low oromotor tone, and sleepy behavior. As a result, late preterm infants have an earlier onset of exhaustion during meals and an earlier cessation of breastfeeding. In this complex scenario, it must be considered that the feeding difficulties experienced by late preterm infants may be exacerbated by the need for admission to neonatal intensive care, which causes early separation from their mothers. In addition, gestational age is inversely correlated with the chance of experiencing eating issues. Remarkably, feeding difficulties appear to negatively impact the mother’s mental health and the mother–infant relationship, resulting in anxiety, stress, and depression.

These data strongly underline the need for more attention and support by healthcare professionals who play a leading role in the promotion and support of breastfeeding and the education and support of mothers, responding to their concerns and fears and helping them understand the physiological prematurity of their infants [31,32,33]. Indeed, an awareness of the diversity of breastfeeding experience may contribute to providing tailored professional care and supportive relationships [34,35]. Lau et al. [36] highlighted the need to consider the specific interplay of the several factors that may impact breastfeeding within the individual maternal-infant dyads when planning interventions to promote and support positive breastfeeding experiences. Breastfeeding late preterm infants is particularly challenging due to the influence of several factors related to the prematurity status (i.e., sleeping behavior, low oromotor tone). For this reason, healthcare breastfeeding support that considers the strict interrelationship between prematurity and feeding behaviors has been strongly advocated [37].

Ravn et al. [38] reported that an early intervention to promote the establishment of good interaction between infants and mothers contributes to the reduction of postpartum depression and successful breastfeeding. Accordingly, Park et al. [39] demonstrated that mothers of very preterm infants, when showing psychological distress, are less prone to have developmentally supportive feeding behaviors. These results indicate that targeted intervention to support maternal psychological well-being may be useful in promoting early feeding interactions within the dyad. Remarkably, Rosenblad et al. [40] reported that mothers of preterm infants who gained high scores on the Breastfeeding Self-efficacy Scale-Short Form at term perceived their infants as having better state regulation at three months. This finding is noteworthy not only because maternal self-efficacy can be strengthened by targeted intervention resulting in a better breastfeeding outcome, but also because greater maternal self-efficacy facilitates better interaction and mutual adaptation within the dyad.

Numerous authors [20,22,27,28,29,30] have claimed that feeding difficulties impair the mother’s mental health and the emotional bond between the mother–infant dyad, resulting in anxiety, stress, and depression. Maternal stress may be responsible for several hormonal changes, which may result in decreased milk supply and, on the other hand, impaired maternal behavior, a barrier to successful breastfeeding [27,28,33,34].

Specifically, it is acknowledged that maternal stress and concerns for the baby negatively affect breastfeeding due to the inhibitory effect of stress on oxytocin and prolactin secretion [33]. Moreover, reduced milk production leads to a feeling of failure in the mother, thus negatively affecting breastfeeding success. Maternal well-being is crucial for successful breastfeeding initiation and establishing a harmonious dyadic relationship [29,40]. Indeed, an altered maternal psychological status can be associated with a major difficulty in adequately perceiving neonatal feeding cues considering the preterm infants’ immature behavior, which frequently lacks the common early signs of hunger [14,39,41].

Both the maternal psychological discomfort and the immature behavior of late preterm infants could hinder the interaction of the dyad, thus favoring a conflictual approach during feeding [28,41,42,43]. Salvatori et al. [42] showed that preterm dyads had fewer positive interactions during feeding times at 18 and 24 months than term dyads; moreover, preterm infants had increased food refusal behaviors from 18 months to 24 months. In line with these findings, Yatziv et al. [43] showed that mothers of preterm infants experienced higher emotional distress than full-term mothers, were less responsive to their infant’s hunger cues, showed a more intrusive attitude, and left their infants less autonomy feeding than mothers of term ones. These feeding challenges can lead to increased stress and anxiety for mothers. The pressure to ensure adequate nutrition and weight gain for their late preterm infants, combined with the demands of triple feeding, can take a toll on mothers’ mental health. Additionally, mothers may feel overwhelmed by the need to monitor their infants’ progress closely and adhere to strict feeding schedules.

Mothers’ mental health is crucial for the overall well-being of both the mother and the infant. Poor mental health can manifest in various ways, including sleep disturbances and exhaustion. When a mother’s mental health suffers, it can negatively impact her ability to care for her infant, further exacerbating the challenges associated with feeding late preterm infants. Furthermore, the post-discharge period can be a particularly challenging time for mothers of late preterm infants. The transition from the hospital to the home environment can be stressful, and mothers may not have the same level of support from healthcare professionals as they did during their hospital stay. This lack of support can increase feelings of isolation and anxiety, contributing to a mother’s mental health deterioration.

Increasing evidence indicates that maternal mental health problems not only affect the mother-to-infant bonding and breastfeeding success but also track the child’s emotional, psychosocial and neurofunctional development. Studies mainly conducted in low-income countries suggest a negative effect of maternal postpartum depression on infants’ weight gain. These negative outcomes could be partly mediated by the infants’ dissatisfaction with the relationship with their mothers, which is largely influenced by the quality of the early mother–infant relationship [44,45]. In light of this long-lasting effect, it is important to support and educate mothers of late preterm infants in providing effective and responsive caregiving behaviors, including the capacity to understand specific feeding cues of late preterm infants. As a result, maternal confidence related to the care of their infant could be enhanced, leading to an improvement of the relationship within the dyad and the related long-term outcomes [46].

Our review presents at least some limitations. Firstly, we performed a narrative literature review, which is less rigorous than a systematic review and could be affected by potential selection bias. Moreover, we did not review the grey literature. Nonetheless, we decided on a predefined search strategy, including multiple databases.

This study emphasized the high prevalence of feeding difficulties in late preterm infants, the importance of implementing targeted support interventions to promote breastfeeding success during the hospital stay and after discharge, and the establishment of harmonious dyadic interaction between the mother and her infant all of which contribute to the prevention of altered feeding behavior later in life. Based on the present findings, healthcare professionals should be properly trained in the appropriate management of breastfeeding in late preterm infants to support mothers in achieving successful breastfeeding. The provision of adequate breastfeeding support associated with promotion of maternal well-being and improved access to perinatal mental health care services is strongly recommended to make mothers feel safe and prone to communicate the difficulties, stress, and anxiety they are experiencing in taking care of their late preterm infant, including feeding. Moreover, promoting and supporting breastfeeding and maternal mental health positively affect mother–infant bonding. The feeding difficulties experienced by late preterm infants can have a significant impact on the mental health of their mothers, particularly during the post-discharge period. Addressing these challenges requires a multifaceted approach, including education, emotional support, and access to mental health resources. By prioritizing the mental health of mothers, healthcare providers and support networks can help to ensure the well-being of the dyad.

There is still a need for additional research to develop a standardized and shared strategy that has been proven to be effective. Further studies with robust methodological designs are needed to elucidate the interventions that might prove most useful for accompanying mothers through their breastfeeding journey while promoting the well-being of the mother and the dyad. Should this be accomplished, it will be possible to offer appropriate support for mothers, encourage the oral skills and maturation of late preterm infants, and improve the relationship quality within the dyad.

## Figures and Tables

**Table 1:** Summary of the included studies about the occurrence of feeding difficulties in late preterm infants and the effect of feeding difficulties on maternal mental health and maternal-infant relationship.

Study	Type of Study	Population	Principal Aim	Main Findings
Giannì et al. 2016 [13]	Cross-sectional questionnaire survey	92 mothers of late preterm infants admitted to level I and II of care	Identification of the facilitators and barriers to breastfeeding during hospitalization according to the experience of mothers	Any human milk feeding at discharge: 94% Exclusively human milk feeding at discharge: 43%
Dosani et al. 2016 [14]	Exploratory mixed method study	122 mothers of late preterm infants (quantitative data) 11 late preterm mothers and 10 public health nurses (qualitative data)	Assessment of breastfeeding rate Assessment of maternal breastfeeding experience Assessment of breastfeeding perception by public health nurses	Any breastfeeding at 6–8 weeks postpartum: 82% Exclusively breastfeeding14% Mothers experienced significant difficulties in breastfeeding their infants
Kair et al. 2015 [15]	Qualitative study	44 late preterm mothers	Assessment of maternal breastfeeding experience at weaning	Breastfeeding was a positive bonding experience Mothers reported milk supply concerns, negative experiences with breast pumping, and feelings of failure.
Crippa et al. 2019 [16]	Prospective observational study	189 late preterm infants admitted to level I of care	Identification of variables affecting breastfeeding duration through the first three months of life	Exclusively breastfeeding rate at discharge: 16.8% Exclusively breastfeeding rate at 15 days: 40.3% Exclusively breastfeeding rate at 40 days: 33.8% Exclusively breastfeeding rate at 90 days: 31.1%
Nagulesapillai et al. 2013 [17]	Community-based prospective pregnancy cohort	173 late preterm infants and 2278 full-term infants	Comparison of breastfeeding difficulties and exclusive breastfeeding rate between late preterm and term infants	Exclusively breastfeeding rate of late preterm mothers vs. full-term infants at 4 months: 54.76% vs. 64.2 *p* = 0.03) Being born late preterm is an independent risk factor for the occurrence of breastfeeding difficulties related to the baby (OR 1.72, 95% CI 1.24–2.38).
DeMauro et al. 2011 [18]	Prospective study	571 late preterm infants and 319 early preterm infants	Comparison of the incidence of postdischarge feeding dysfunction and hospital/subspecialty visits for feeding problems during the first 12 months of life in late and early-preterm infants	Oromotor dysfunction borderline or high, early preterm vs. late preterm: - 3 mo: 29% vs. 17%, *p* = 0.04 - 6 mo: 10% vs. 8%, *p* = 0.34 - 12 mo 7% vs. 4%, *p* = 0.03) Avoidant feeding behavior, medium or high, early preterm vs. late preterm: - 3 mo: 33% vs. 29%, *p* = 0.04 - 6 mo: 18% vs. 20%, *p* = 0.73 - 12 mo: 14% vs. 12, *p* = 0.77 Hospital/subspecialty visits, early preterm vs. late preterm: - 3 mo: 17% vs. 12%, *p* = 0.11 - 6 mo: 13% vs. 12%, *p* = 0.90 - 12 mo: 17% vs. 12%, *p* = 0.09
Lee et al. 2019 [19]	Observational study	106 late preterm infants admitted to Neonatal Intensive Care Unit	Assessement of breastfeeding rates untile 12 weeks after discharge	1 week: - exclusive breastfeeding 5.7% - mixed feeding 67% 2 weeks - exclusive breastfeeding 7.6% - mixed feeding 52.8% 3 weeks - exclusive breastfeeding 9.4% - mixed feeding 38.7% 4 weeks - exclusive breastfeeding 10.4% - mixed feeding 31.2 8 weeks - exclusive breastfeeding 17% - mixed feeding 16 12 weeks - exclusive breastfeeding 19.8% - mixed feeding 12.3%
McDonald et al. 2013 [20]	Community-based prospective cohort study	77 late preterm and 1150 full-term mothers	Evaluation of the independent effect of late preterm birth on maternal mental health Comparison of breastfeeding rates and health care utilization between late preterm and full-term mothers.	Late preterm mothers were at higher risk of having anxiety symptoms at four months after delivery (OR 2.07, CI 1.08; 3.98) Breastfeeding rates late preterm vs. full-term mothers: - within 24 h after birth: 78.7% vs. 97.5%; *p* < 0.001 - before discharge: 78.1% vs. 90.8%; *p* < 0.001 - at 4 months: 69.3% vs. 81.7%, *p* = 0.008 Health care utilization late preterm vs full-term mothers: 14.5% vs. 3.8%; *p* < 0.001.
Jonsdottir et al. 2020 [21]	Observational cohort study	122 late preterm mothers whose infants were either admitted to maternity ward (LPT MU) or neonatal intensive care unit (LPT NICU) and 269 full-term mothers	Comparison of breastfeeding rates between LPT MU, LPT NICU and term mothers	Breatsfeeding initiation at the hospital: - 90% LPT NICU vs. 100% LPT MU *p* < 0.05 Exclusive breastfeeding during the 1st week - 63% LPT NICU and 56% LPT MU vs. 86% term infants *p* < 0.01 Exclusive breastfeeding at 1 mo - 58% LPT NICU and 53% LPT MU vs. 77% term infants *p* < 0.01
Jonsdottir et al. 2021 [22]	Longitudinal cohort study	129 late preterm mothers and 277 full-term mothers	Comparison of breastfeeding rates at 1, 4, 8, 12 mo between late preterm and full-term mothers Comparison of maternal well-being at 1, 4, 8, 12 mo between late preterm and full-term mothers.	Decline in exclusive breastfeeding from 1 to 4 months: - 15% for late preterm infants (chi-square 5.37, *p* < 0.05) - 31% for term infants (chi-square 49.84, *p* < 0.001) Median estimated breastfeeding time: 7 months (95% CI 5.53–8.48) for late preterm vs. 9 months (95% CI 8.39–9.61) for full-term infants Late preterm vs. full-term mothers worries about infant’s nutrition, - at 1 month, 53% vs. 33% ns - at 4 months, 44% vs. 34% (*p* = 0.07). - at 12 months 44% vs. 39%, ns Edinburgh Postnatal Depression Score ≥ 13: at 1 mo: 12% vs. 9%, ns at 4 mo: 18% vs. 8%, (*p* < 0.01) Total Parenting Stress Index scores or the scores for the subscales at 12 mo: ns
Medoff Cooper et al. 2012 [23]	Multicenter prospective study	802 late preterm infants	Description of the neonatal risks including the occurrence of feeding difficulties	Feeding difficulties occurred in 40.6% of infants: 61% in infants born at 34 weeks vs. 42% and 35% in infants born at 35 and 36 weeks
Hellmeyer et al. 2012 [24]	Single-center retrospective cohort study	893 late preterm infants	Assessment of the nature and frequency of neonatological complications	Feeding difficulties - 60% in infants born at 34 weeks vs. 50% infants born at 35 weeks vs. 29.10% in infants born at 36 weeks (*p* < 0.001).
Gianni et al. 2015 [4]	Single-center retrospective study	1768 late preterm infants	Assessment of the need of nutritional support during hospital stay	592 infants required a nutritional support; 2.6% required tube feeding: 5.3% infants born at 34 weeks, 2.8% infants born at 35 weeks 1.3%, infants born at 36 weeks
Lau et al. [25]	Observational study	48 late preterm infants	Assessment of the maturity levels of oral feeding skills at the time of first oral feeding	Most immature oral feeding skills: 18.7% in infants born at 34 weeks vs. 10.4% in infants born at 35 weeks *p* = 0.035
Demirci et al. 2013 [26]	Population-based cohort study	68.886 late preterm infants, 17.325 moderately preterm infants, 870.034 full term infants	Comparison of breastfeeding initiation rates among late preterm with moderately preterm and full term mothers (2003–2009)	Late preterm infants breastfeeding initiation increased from 54% to 61.8% (*p* < 0.001). The breastfeeding initiation in cumulative years 2003–2009 was greater among term infants (uOR 1.44, 95% CI 1.42–1.46, *p* < 0.001) - Compared to mothers of 34 week infants, mothers of 35- and 36-week infants were more likely to initiate breastfeeding (respectively: uOR 1.08, 95% CI 1.03–1.13, *p* < 0.01; uOR 1.14, 95% CI 1.09–1.19, *p* < 0.001).
Zanardo et al. 2011 [27]	Prospective case control study	42 late preterm and 42 full-term mothers	Assessment of maternal psychological distress on day 3–4 postpartum	Late preterm vs. full-term mothers: - trait anxiety 45.8 ± 10.1 vs. 39 ± 6.1, *p* < 0.02; - state anxiety 49.5 ± 9 vs. 42.6 ± 5.3, *p* < 0.002; - Edinburgh Postnatal Depression Scale: 9.5 ± 4.5 vs. 6.3 ± 3.9, *p* < 0.0008; - Psychological Stress Measure: 46.5 + 5.9 vs. 38.9, *p* < 0.001)
Zanardo et al.2017 [28]	Prospective case–control study	30 late preterm and 60 full-term mothers	Comparison of the personality profile and attitudes toward lactation between late preterm and full-term mothers	Late preterm vs. full-term mothers: Mother-to-Infant Bonding Scale (mean ±SD scores): 1.364 versus 0.581, *p* = 0.026 Mother-to-Infant Bonding Scale (Subscales mean ± SD scores) - Disappointed: 0.33 ±0.80 vs. 0.05 ±0.21 *p* = 0.012 - Dislike 0.26 ± 0.58 vs. 0.03 ± 0.18 *p* = 0.005 Luscher Color Test general interpretation: late preterm mothers developed deep stress
Gerhardsson et al. 2020 [29]	Longitudinal prospective study	105 late preterm mothers	Assessment of the association between mothers’ self- efficacy and their adaptation to the late preterm breastfeeding behavior.	a 1 point higher at Breastfeeding Self Efficacy Scale was associated with a 0.169 point higher score at the Adaptation to the Late Preterm Infant when Breastfeeding Scale (*p* < 0.001)
Gerhardsson et al. 2018 [30]	Prospective, comparative study	148 late preterm mothers at their infants’ 40 weeks of postmenstrual age 114 late preterm mothers at their infants’ 3 mo of corrected age	Assesment of self-efficacy in breastfeeding	Breastfeeding Self-Efficacy Scale scores at 40 weeks: - Mothers exclusively breastfeeding vs. mothers partially breastfeeding or not breastfeeding at hospital discharge: 57.5 vs. 54, *p* = 0.05 - Mothers exclusively breastfeeding vs. mothers partially breastfeeding or not breastfeeding at discharge: 58.6 vs. 47.9, *p* < 0.001 - Mothers exclusively breastfeeding vs. mothers partially breastfeeding or not breastfeeding at 40 weeks: 57.1 vs. 41.4, *p* < 0.001

## Data Availability

Data sharing not applicable.
